# BarleyExpDB: an integrative gene expression database for barley

**DOI:** 10.1186/s12870-023-04193-z

**Published:** 2023-03-31

**Authors:** Tingting Li, Yihan Li, Hongbin Shangguan, Jianxin Bian, Ruihan Luo, Yuan Tian, Zhimin Li, Xiaojun Nie, Licao Cui

**Affiliations:** 1 College of Bioscience and Engineering, Jiangxi Agricultural University, Nanchang, 330045 Jiangxi China; 2 State Key Laboratory of Crop Stress Biology in Arid Areas and College of Agronomy, Northwest A&F University, Yangling, 712100 Shaanxi China; 3 Peking University Institute of Advanced Agricultural Sciences, Weifang, 261325 Shandong China; 4 Xintai Urban and Rural Development Group Co., Ltd, Taian, 271200 Shandong China

**Keywords:** Barley, Expression, RNA-sequencing, Database, BarleyExpDB

## Abstract

**Background:**

RNA-sequencing (RNA-seq) has been widely used to study the dynamic expression patterns of transcribed genes, which can lead to new biological insights. However, processing and analyzing these huge amounts of histological data remains a great challenge for wet labs and field researchers who lack bioinformatics experience and computational resources.

**Results:**

We present BarleyExpDB, an easy-to-operate, free, and web-accessible database that integrates transcriptional profiles of barley at different growth and developmental stages, tissues, and stress conditions, as well as differential expression of mutants and populations to build a platform for barley expression and visualization. The expression of a gene of interest can be easily queried by searching by known gene ID or sequence similarity. Expression data can be displayed as a heat map, along with functional descriptions as well as Gene Ontology, Kyoto Encyclopedia of Genes and Genomes, Proteins Families Database, and Simple Modular Architecture Research Tool annotations.

**Conclusions:**

BarleyExpDB will serve as a valuable resource for the barley research community to leverage the vast publicly available RNA-seq datasets for functional genomics research and crop molecular breeding.

**Supplementary Information:**

The online version contains supplementary material available at 10.1186/s12870-023-04193-z.

## Background

Over the last decade, RNA-sequencing (RNA-seq) has surpassed microarray to become the common technique in biological studies [1]. It is a powerful analytical tool for transcriptional profiling to study gene structures, splicing patterns, and gene/isoform expression levels [2]. The proliferation of the use of next-generation sequencing technologies in the plant research community has led to the accumulation of copious amounts of RNA-seq data in different plant species [3]. By sequencing RNA samples collected from different parts of the plant or plants cultivated under different conditions, such as tissues, developmental stages, and biotic and abiotic stress treatments, the hypotheses of the functions of specific genes/isoforms can be determined [4]. These approaches have been widely used in functional studies aimed at uncovering regulatory mechanisms in major crops such as maize, wheat, and rice [5].

Numerous studies have generated large volumes of raw sequencing data that have been deposited in online repositories such as the Sequence Read Archive (SRA) [6], European Nucleotide Archive (ENA) [7], and Genome Sequence Archive (GSA) [8]. By 2021, these archives have collectively released a total of ~ 45,000 libraries for major crops, including wheat, rice, maize, and cotton [9]. Retrospective analyses of the massive amount of RNA-seq data can accelerate functional genomics research and illuminate biological insights [10, 11]. However, these data are available as data archival repositories that store raw sequencing reads, whose access is costly for many academic groups that lack specialized computational resources or dedicated bioinformatics personnel [1].

Efforts have been made to standardize and simplify the access to gene expression data generated by RNA-seq to create a unified resource from fragmented repositories [12]. Recently, comprehensive online databases were established to enhance the utilization of these RNA-seq datasets. For example, the *Arabidopsis* RNA-seq (ARS) database provides a comprehensive platform with integrated, user-friendly, and multifaceted functions for exploring *Arabidopsis* RNA-seq libraries [1]. WheatExp provides free access to a comprehensive array of expression data which allows users to decipher homologue-specific gene expression profiles across a broad range of tissues from different developmental stages in polyploid wheat [13]. BnTIR was established using comprehensive RNA-seq datasets from 91 different tissues spanning *Brassica napus* development [14]. Robinson et al. developed a quick and easy-to-use platform (AgriSeqDB) for RNA-seq data visualization and analysis in *Arabidopsis* and five major crops [5]. Yu et al. constructed the Plant Public RNA-seq Database (PPRD) for viewing, analyzing, and interpreting different mutants, tissues, developmental stages, and stress conditions from several species [9]. These resources have become increasingly valuable exploratory tools for deciphering the complex architecture of the regulatory mechanisms that govern biological processes in diverse species.

As one of the prominent crops since the dawn of early agricultural civilization, cultivated barley (*Hordeum vulgare* L. ssp. *vulgare*) is mainly used for feeding animals, malting, and brewing [15]. Barley is one of the most adaptable plants that can withstand harsh conditions better than its close relative wheat, and it maintains an important role in human nutrition in areas with harsh climates [16]. Wild barley (*H. vulgare* L. ssp. *spontaneum*), domesticated ca. 10,000 years ago in the Fertile Crescent, is the progenitor of cultivated barley and serves as a rich genetic resource for barley improvement [17]. As another important variety of barley, Tibetan hulless barley (*H.vulgare* L. var. *nudum*) is the principal cereal cultivated by Tibetans and a key livestock feed in the Tibetan Plateau [18, 19]. Similar to that in other crops, genomics has been a major driver of genetic and breeding advances in barley over the past decade [15]. The barley genomic assemblies, including the first draft genome and its subsequent revisions (Morex V1, V2, and V3), have undergone multiple rounds of refinement with the emergence of computing algorithms (such as TRITEX workflow) and new sequencing technologies (such as PacBio HiFi, 10 × genomics, chromosome conformation capture sequencing (Hi-C), and biological nano optical mapping) [20–22]. The recently released barley genome and pan-genome expanded the range of natural or induced sequence variation to facilitate genetic analysis and breeding [15, 19, 23]. Benefiting from the release of the genome, copious amounts of RNA-seq-based transcriptome data have been produced and are available for comparative analysis [24–26]. To support sharing and utilizing, researchers have constructed various repositories for RNA-seq datasets of barley [27–29]. However, these existing databases are not well integrated with the currently published RNA-seq datasets, especially in the mining of expression profiles of wild barley and Tibetan hulless barley. In addition, the unsupported reference genome, with the most updated version, is also inconvenient to use. Therefore, a large-scale database with comprehensive RNA-seq datasets that can provide visualized transcriptome expression patterns for barley is greatly needed.

**Fig. 1:**
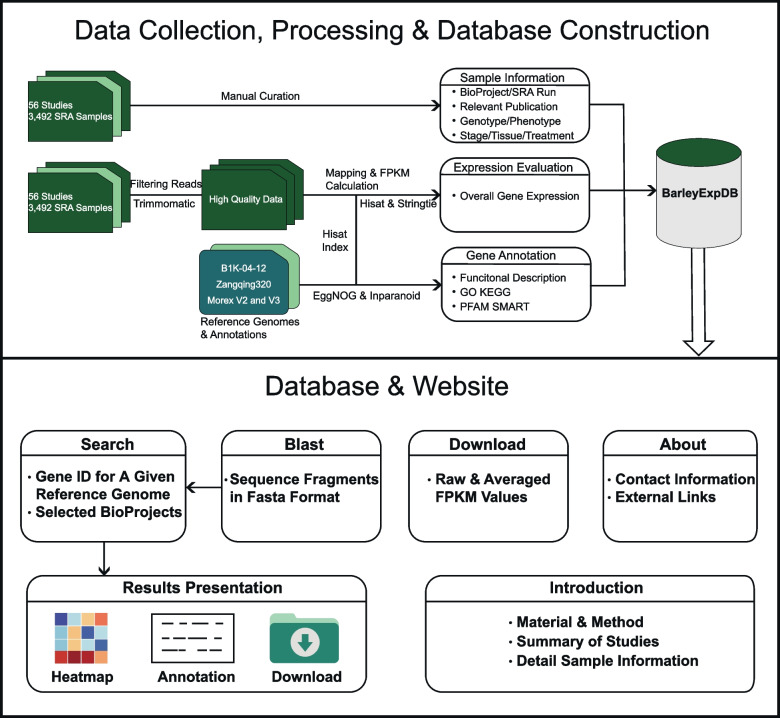
Workflow of BarleyExpDB

Here, we construct the barley expression database (BarleyExpDB: http://barleyexp.com/) (Fig. 1), a web-accessible resource that integrates 56 studies consisting of 3,492 publicly available barley RNA-seq library data deposited at the National Center for Biotechnology Information (NCBI) Sequence Read Archive (SRA). BarleyExpDB supports gene ID queries for four different barley genomes, including cultivated barley (Morex V2 and V3), wild barley (B1K-04-12), and Tibetan hulless barley (Zangqing320), to improve their viability and functionality. In addition, the database provides a user-friendly interface to efficiently visualize, organize, and download the expression profiles of different subspecies/varieties, mutants, stages/tissues, and stress treatments, as well as recombinant inbred lines (RIL) and near isogenic lines (NIL) population. With the rapid growth of barley RNA-seq libraries and the continuous improvement of the reference genome, we plan to regularly update BarleyExpDB in the future. Our approach is designed to provide free, user-friendly, and comprehensive expression data to support researchers in gaining new biological insights and generating new hypotheses in molecular evolution and breeding.

### Construction and content

#### RNA-seq data collection and data processing

A total of 56 studies comprised of 3,492 RNA-seq libraries were included in BarleyExpDB (Supplementary Table S1). These datasets maximally represent the barley expression datasets across multiple subspecies/varieties, developmental stages/tissues, mutants, and stress treatments, as well as RIL and NIL populations. The RNA-seq datasets were downloaded from the NCBI SRA database using the prefetch option in SRAtoolkit v2.10.8. The downloaded SRA files were transformed into FASTQ files using the parallel-fastq-dump tool (github.com/rvalieris/parallel-fastq-dump). The quality control of raw reads was performed using Trimmomatic v0.36 with the following parameter options: Minlen of 90, Trailing of 3, Leading of 3, and SlidingWindow of 4:5.

The barley Morex V2, V3, and B1K-04-12 reference assemblies were downloaded from the IPK database (https://doi.org/10.5447/ipk/2019/8, http://doi.org/10.5447/ipk/2021/3, and https://doi.ipk-gatersleben.de/DOI/c4d433dc-bf7c-4ad9-9368-69bb77837ca5/3490162b-3d76-4ba1-b6ee-3eaed5f6b644/2). The reference genome of Zangqing320 was retrieved from WheatOmics (http://wheatomics.sdau.edu.cn/). Hisat v2.1.0 was used to build the index for the genomic assembly and to align the RNA-seq reads onto the reference genome. SAM files were converted into BAM format and then the BAM files were sorted using the ‘bS’ and ‘sort’ options of SAMtools v1.3.1. Stringtie v1.3.5 was used for calculating the fragments per kilobase of transcript per million mapped reads (FPKM) values for each gene.

### Database implementation and web interface

The web server is hosted on the Tencent Cloud’s lightweight application server with four Intel(R) Xeon(R) Platinum 8255C CPUs at 2.50 GHz with 8 GB of RAM and can be freely accessed through its website for non-commercial use. The server uses the Linux CentOS v7.9 operating system (http://www.centos.org). The front-end web interface was developed using HTML (https://www.w3.org/html/), JavaScript (https://www.javascript.com/), and CSS (http://www.w3.org). The server-side back-end was implemented by PHP (https://www.php.net/). Gene expression matrix storage, maintenance, and operation are supported by MySQL v5.6.50. The custom PHP code was written to enable data searches from MySQL, which were transferred to the front-end.

### Development of data mining tools

Functional descriptions, Kyoto Encyclopedia of Genes and Genomes (KEGG) pathways, Gene Ontology (GO) terms, enzyme commission (EC) numbers, Protein Families Database (PFAM) designations, and Simple Modular Architecture Research Tool (SMART) protein domains were annotated using eggNOG-mapper v2 (http://eggnog-mapper.embl.de/) [30]. The Basic Local Alignment Search Tool (BLAST) was implemented using ViroBlast, a standalone BLAST web server, for sequence homology searches [31]. Orthologs to rice (https://oct2017-plants.ensembl.org/Oryza_sativa/Info/Index) and *Arabidopsis thaliana* (https://www.arabidopsis.org/) were identified using Inparanoid v8.0. The interactive heatmap was rendered using Plotly.js (Plotly Technologies Inc., Collaborative Data Science, Montréal, QC, 2015. https://plotly.com/) with specified versatile parameters and exported in PNG format.

### Utility and discussion

#### Data quality control and initial mapping statistics

**Fig. 2:**
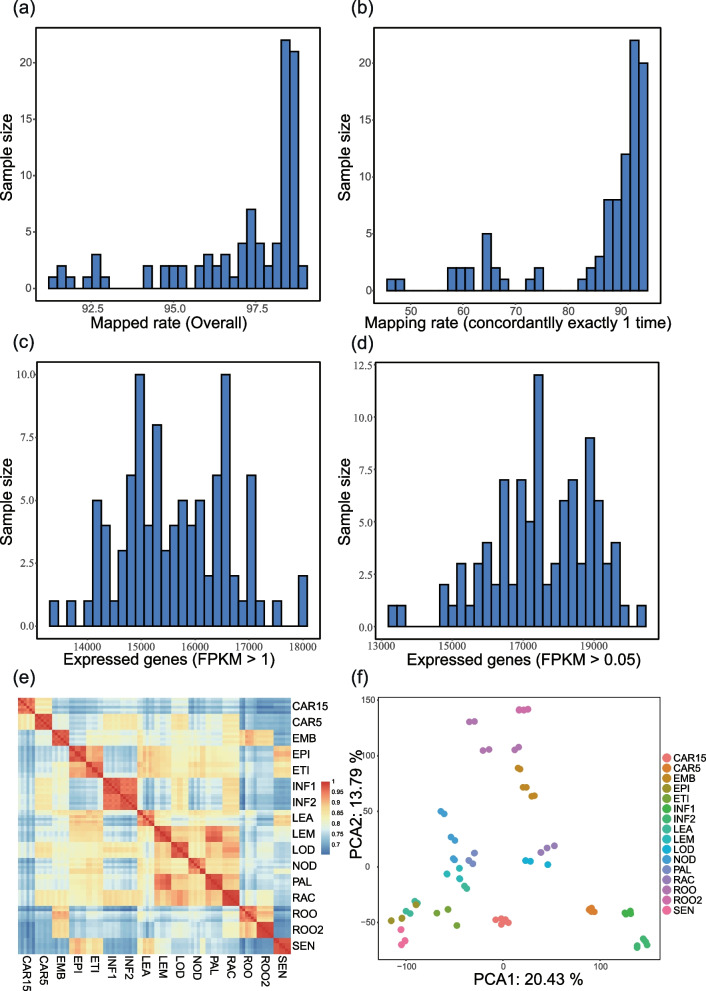
Landscape of the mapping statistics and expression profiles (e.g., PRJEB14349: RNA-seq of 16 developmental stages of barley). **a** Overall mapping rate. **b**. Concordantly exactly one time mapping rate. **c**. Gene number (FPKM > 1) distribution. **d**. Gene number (FPKM > 0.05) distribution. **e**. Heatmap of pair-wise Pearson coefficients between samples. **f**. Data representation by principal component analysis for all samples

To evaluate the data quality, the sequence quality, GC content, and mapping rate were estimated for each sample. A total of over 12 TB of high-quality clean data were generated using the commonly endorsed criteria of Trimmomatic v0.36. The alignment summary revealed that most reads were aligned concordantly exactly one time to the reference genome, which supported the reliability of the RNA-seq datasets (Fig. 2a-d).

### Reproducibility of biological samples

The reproducibility of the gene expression profiles across biological and technical replicates was further used to evaluate the quality of the RNA-seq datasets. The pair-wise Pearson correlation coefficient between any two of the samples within the same sample group was calculated based on all genes in the barley genome. Taking the BioProject PRJEB14349 as an example, the mean correlation coefficient value and the standard error per sample group were 0.8069 and 0.1368, respectively, suggesting a high level of sample reproducibility and measurement consistency among replicates (Fig. 2e). Principal component analysis (PCA) confirmed these findings and revealed a strong correlation between replicates of the same stage/tissue, but a significantly lower correlation between samples from different stages/tissues (Fig. 2f).

### Comparison with published research

Validation experiments, such as qRT-PCR, were not performed because germplasm resources and sample materials were not available in the corresponding studies. However, existing studies have confirmed the high degree of uniformity among biological replicates of the same stage/treatment as well as the distinctness between stages/treatments by PCA [32–34], hierarchical clustering [34], and multidimensional scaling (MDS) analysis [35]. In addition, qRT-PCR validation of the candidate genes was performed, and the results showed high correlation coefficients with transcriptomic expression levels, confirming the reliability of the transcriptome analysis [25, 32, 34, 36]. Notably, several researchers have performed in-depth experiments, such as overexpression and virus-induced knockdown, to verify the biological functions of candidate genes [25, 37]. Taken together, these findings indicated that the RNA-seq data were valid. We conclude that BarleyExpDB is a valuable resource for the research community to efficiently utilize the vast publicly available RNA-seq datasets, with biological functions and molecular mechanisms to be further investigated by researchers.

### Database implementation and practical tools

**Fig. 3:**
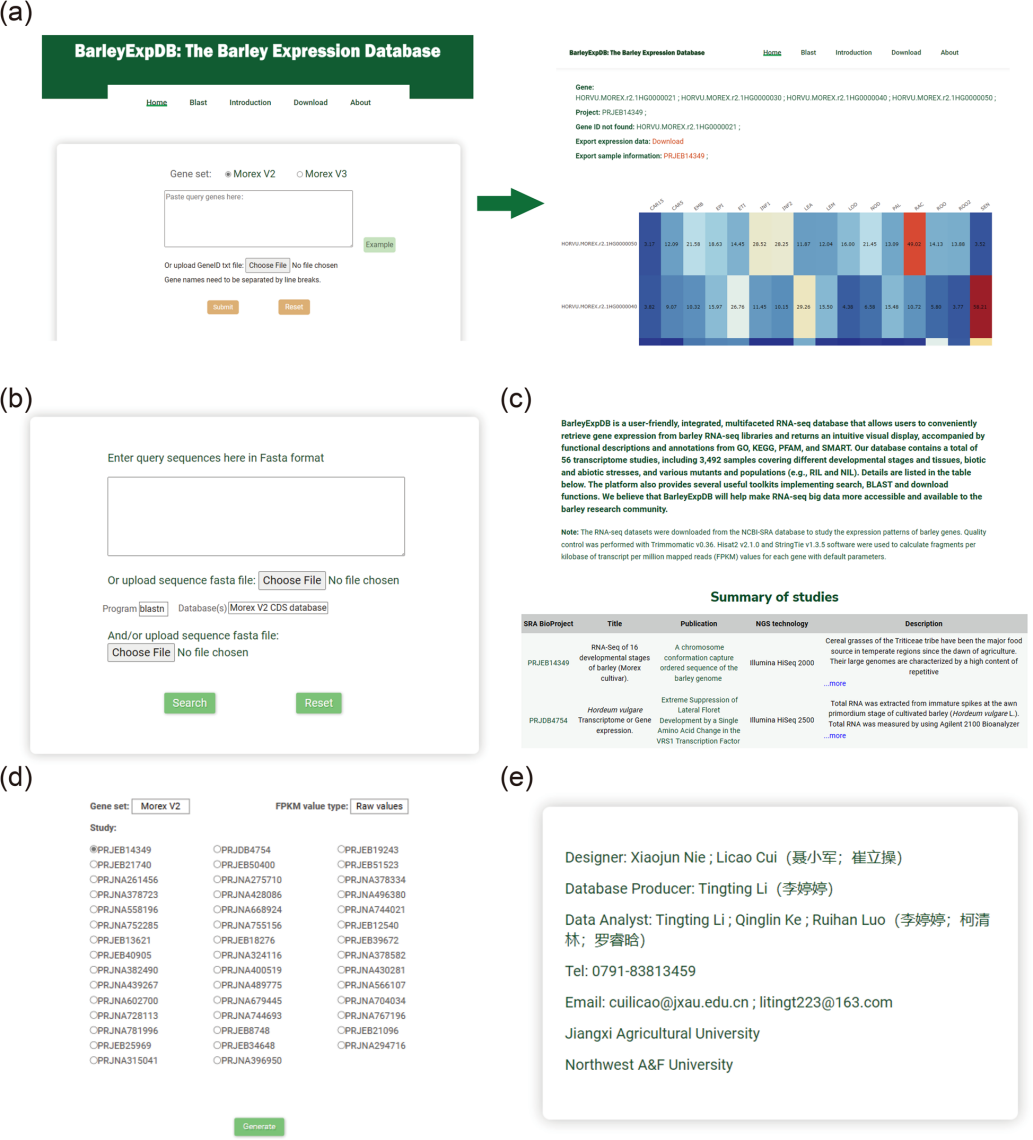
Overview of BarleyExpDB. **a** Homepage of BarleyExpDB and example results. **b**. Sequence similarity search tools. **c**. Introduction of BarleyExpDB. **d**. Downloaded pages. **e**. Contact information and external links

The BarleyExpDB is publicly accessible through the easy-to-use and intuitive web link http://barleyexp.com/. The web interface contains five main sections, namely, Home, BLAST, Introduction, Download, and About (Fig. 3).

#### Home

The home page of BarleyExpDB provides three main modules for cultivated barley (reference genome: Morex V2 and V3), wild barley (B1K-04-12), and Tibetan hulless barley (Zangqing320). Each module provides a brief descriptive list of studies currently available in our database, as well as a user-friendly search box for gene expression profile queries. From this main hub, users should select the corresponding BioProject studies according to their individual research needs. Notably, we provide a tag for each study that includes summary information to make it easy for users to quickly select the study they are interested in. The search function of the BarleyExpDB can be queried in one of the four gene sets, such as Morex V2 (e.g., HORVU.MOREX.r2.1HG0000021) [22], Morex V3 (e.g., HORVU.MOREX.r3.1HG0000030) [38], B1K-04-12 (Horvu_FT11_1H01G003300) [15] or Zangqing320 (MLOC_42) [19]. Users can query the genes of interest directly in the search box. For larger-scale analyses, it is recommended that the user provide a processed text file with one gene ID per line and optionally upload it to BarleyExpDB. The maximum single query allowed in the database is 500 genes at a time. If more query genes are needed, users can query by submitting in batches, or directly download the raw data and extract the files by themselves.

The graphical representation profiles of all datasets are presented on a heat map showing the average RPKM values. Graphics can be downloaded in 'png' image format, and FPKM data is presented in an accompanying table that can be exported in '.csv' format, facilitating the analysis required by end-users. In addition to the expression search tools, we provide comprehensive information on PFAM, SMART, GO, KEGG, and functional descriptions for user-submitted genes, as well as valuable information for the selected RNA-seq study, such as genotype/phenotype, stage/tissue, and relevant publications. It should be of note that users have access to homologous genes in rice and *Arabidopsis thaliana*.

#### Blast

For sequence fragments without definite gene IDs, BarleyExpDB provides an online BLAST service to query across different database collections. Users can submit the amino acid or nucleotide sequences to the search box in Fasta format, or upload them to the database in text file format. Five kinds of BLAST algorithms (e.g., BLASTN, BLASTP, and TBLASTX) can be selected to identify putative homologous sequences. When browsing using the BLAST alignment function, candidate hits are listed in descending order of expectation and are viewed side-by-side in the results window.

#### Introduction

The "Introduction" page provides a brief description of BarleyExpDB and a drop-down menu where users can browse the "Materials and Methods" applied in BarleyExpDB. The analysis software used to build the database can be accessed directly via a link. The commands and parameters are also displayed.

The interface also provides a comprehensive description of each RNA-seq study in BarleyExpDB, such as sample accessions, stages/tissues, treatments, and related publications, which is valuable information to help users select appropriate samples and conduct subsequent studies.

#### Download

All the FPKM values of the expression matrix that are available for downloading and reanalyzing are listed on the 'Download' page.

#### About

The "About" page displays a few generic external links that users can access quickly.

### Prospects

BarleyExpDB is in a continual state of incremental improvement. These resources will contribute to our understanding of the complex structures that control the regulatory mechanisms of biological processes in the barley genome. Furthermore, BarleyExpDB will be implemented with additional features and utilities to better serve the barley research community: (i) The assembly of telomere-to-telomere (T2T) genomic sequences has been recently reported in various plants [39, 40]. However, none of the seven chromosomes are completed from end to end, and a large number of unresolved gaps and missing sequences have been observed in the rDNA, centromere, and sub-telomere regions of Morex V3 assembly [41]. The development of a gap-closed, newly annotated T2T assembly for barley (which will likely be called Morex V4) is planned to be released soon, and this is our subsequent update of the genomic gene set; (ii) Given the widespread species-wide catalog of gene presence/absence variants (PAV), a single reference genomic context is not competent for the assessment of dispensable gene expression. We expect to integrate the Barley Pangenome V1, or the forthcoming Barley Pangenome v2, to provide genomic–transcriptomic companion expression profile datasets, which will provide important information on the functional studies of specific genes; (iii) With the growing repository of barley transcriptome datasets in the public domain, our database will be updated continuously with more newly release RNA-seq samples. The BarleyExpDB framework is highly scalable and can efficiently integrate newly released RNA-seq expression datasets, ensuring that we can achieve at least two updates per year; (iv) RNA editing is a process by which genetic information is modified in the RNA sequence corresponding to its DNA template [42]. The next version of BarleyExpDB is under development to study post-transcriptionally regulated RNA editing sites by incorporating paired DNA–RNA data; (v) The rapid and enthusiastic adoption of full-length, single-cell, and spatial RNA-seq revolutionizing the details of whole-transcriptome studies [43, 44]. The data generated by these novel sequencing technologies will be integrated into BarleyExpDB with a view to reflecting the full spectrum of differentially alternatively spliced transcripts and snapshots from tissue to cell; (vi) New functions and analytical tools will be implemented in BarleyExpDB for the convenience of users, such as identification of tissue-specific genes and online eGWAS prediction of phenotype-related genes. Our approach will maximize the utility of the database in studying different aspects of barley development, enabling researchers and small labs without computing resources to mine complex and valuable expression datasets. We also welcome complementary RNA-seq datasets from third-party research groups to enrich our database.

## Conclusions

The rapid development of next-generation sequencing technologies, coupled with the decreasing cost of sequencing, has led to the accumulation of copious amounts of expression data. We present BarleyExpDB, a convenient, web-accessible, and management-flexible RNA-seq database in barley that allows users to quickly scan the abundant information using the known gene ID of Morex V2, V3, B1K-04–12 and Zangqing320. BarleyExpDB is currently the most comprehensive RNA-seq database in barley and provides the expression levels in various tissues, developmental stages, environmental stresses, as well as in different genotypes, phenotypes, mutants and populations. Our database also implements several practical utilities for sequence homology searching, visualization, function annotation, and result downloading. We believe that BarleyExpDB will contribute to the acquisition and utilization of transcriptome big data and advance functional genomics and breeding biology research in barley.

## Supplementary Information

**Additional file 1:** **SupplementaryTable 1.** Detail sample informationof the RNA-seq datasets downloaded from the NCBI SRA database.

## Data Availability

Data pertaining to the study have been included in the article, and further inquiries can be directed to the corresponding authors. The barley genomes were downloaded from the given links: https://doi.org/10.5447/IPK/2019/19 (Morex V2), http://doi.org/10.5447/ipk/2021/3 (Morex V3), https://doi.ipk-gatersleben.de/DOI/c4d433dc-bf7c-4ad9-9368-69bb77837ca5/3490162b-3d76-4ba1-b6ee-3eaed5f6b644/2 (B1K-04–12), and http://wheatomics.sdau.edu.cn/ (Zangqing320). To comprehensively evaluate the expression pattern of barley, publicly available RNA-seq datasets were obtained from the NCBI SRA database with BioProject numbers PRJNA629999, PRJNA507455, PRJNA748178, PRJNA828098, PRJNA227211, PRJNA432492, PRJNA261456, PRJEB4947, PRJNA679445, PRJNA491382, PRJNA665933, PRJNA489775, PRJNA744021, PRJNA543388, PRJNA728483, PRJEB12540, PRJNA324116, PRJNA400519, PRJNA439267, PRJNA546269, PRJNA566107, PRJNA602700, PRJEB40905, PRJNA704034, PRJNA728113, PRJNA744693, PRJEB39672, PRJNA496380, PRJNA428086, PRJEB14349, PRJNA378582, PRJNA558196, PRJEB19243, PRJNA294716, PRJNA315041, PRJNA378334, PRJEB25969, PRJEB21740, PRJDB4754, PRJNA396950, PRJNA378723, PRJNA430281, PRJNA275710, PRJEB8748, PRJEB18276, PRJNA382490, PRJEB13621, PRJEB21096, PRJEB34648, PRJEB50400, PRJEB51523, PRJNA668924, PRJNA752285, PRJNA767196, PRJNA781996 and PRJNA755156. The gene expression matrix containing the raw and averaged FPKM values across 56 studies can be directly downloaded from http://barleyexp.com/download.html.

## Abbreviations

ARS: Arabidopsis RNA-seq
BLAST: Basic Local Alignment Search Tool
EC: Enzyme Commission
ENA: European Nucleotide Archive
FPKM: Fragments Per Kilobase of Transcript Per Milliom Mapped Reads
NIL: Near Isogenic Lines
GO: Gene Ontology
GSA: Genome Sequence Archive
KEGG: Kyoto Encyclopedia of Genes and Genomes
MDS: Multidimensional Scaling
NCBI: National Center for Biotechnology Information
PAV: Presence/Absence Variant
PAFM: Proteins Families Database
PCA: Principal Component Analysis
PPRD: Plant Public RNA-seq Database
RIL: Recombinant Inbred Lines
RNA-seq: RNA-sequencing
SMART: Simple Modular Architecture Research Tool
SRA: Sequence Read Archive
T2T: Telomere-to-Telomere
